# Characteristics of Traumatic Lower Extremity Amputation: Medical and Social Comorbidities

**DOI:** 10.7759/cureus.100136

**Published:** 2025-12-26

**Authors:** Mark D Marino, Sophia Izhar, Heidi Israel, Patrickson Jean, John T Watson

**Affiliations:** 1 Orthopaedic Surgery, Saint Louis University School of Medicine, Saint Louis, USA

**Keywords:** diabetes mellitus, hypertension, lower extremity amputation, lower extremity trauma, motor vehicle accident, substance abuse, traumatic amputation, traumatic brain injury

## Abstract

Background

Traumatic amputations are life-altering injuries that remain prevalent despite the best efforts of physicians. While a multitude of research demonstrates various medical comorbidities that contribute to amputations, little attention has been paid to specific factors linked to traumatic amputation, even though trauma is the second most common etiology of amputation. The aim of this study was to determine whether any underlying medical conditions previously associated with non-traumatic amputation in the literature may also be associated with increased rates of amputation in cases of lower extremity trauma.

Methods

A retrospective database was constructed by accessing billing records at a level I trauma center in the United States between 2017 and 2022, identifying 63 cases of traumatic lower extremity amputation. A control group of 129 similar lower extremity fracture patients who did not undergo amputation was isolated from the same period using stratified sampling. The control group was matched to the amputation group in regard to age, sex, race, mechanism of injury, and whether the fracture created an open wound. Pearson chi-square statistical tests were performed to evaluate significant relationships at a 95% confidence interval.

Results

Patients with lower extremity trauma who presented with diabetes, hypertension, osteomyelitis, fasciotomy, alcohol intoxication, and chronic kidney disease were associated with increased rates of amputation. This correlation was preserved even after patients who underwent amputation within the first 48 hours of injury were excluded. Trauma patients who sustained traumatic brain injuries were significantly less likely to require amputation (p = 0.011). Among amputees, patients under the influence of alcohol, marijuana, or opioids/fentanyl were significantly more likely to sustain high-energy polytrauma and incur lower extremity trauma from motor vehicle accidents.

Conclusions

A multitude of medical comorbidities with causal links to chronic amputations were also associated with traumatic lower extremity amputations in this single-center case-control study. Patients who sustained traumatic brain injuries were less likely to require amputation, validating previous research linking head trauma to improved tissue healing or another protective mechanism. Alcohol intoxication was associated with increased rates of traumatic amputation, and associations were observed between substance use and higher severity injuries among amputees.

## Introduction

Amputation resulting from trauma is a life-altering surgical procedure, which is usually performed under great duress, not only for the surgeon but for the patient and the entire operative team as well. Amputations permanently affect a patient’s physical, financial, emotional, and social health [1]. The most common etiologies of amputation include vascular disease (54%), and trauma (45%), which is the second leading cause of amputation, estimated at over 30,000 cases every year [2]. Therefore, it is imperative to understand the factors that contribute to traumatic injuries resulting in amputation. Multiple classification systems have been developed to stratify the prognosis and clinical management of patients with open fractures that threaten the viability of limb salvage [3]. Identifying factors associated with acute amputations will aid physicians in making timely decisions regarding limb salvage and amputations. 

Copious research has linked diabetes, vascular disease, chronic kidney disease (CKD), and socioeconomic factors to increased amputation rates. However, the bulk of this research studies non-traumatic amputations. One recent retrospective study found that diabetics with osteomyelitis were four times more likely to require lower extremity amputation than those with soft tissue infection alone [4]. Rigorous analysis also links microvascular disease (MVD) to general amputations; a combined cohort of patients with peripheral artery disease and MVD was found to have a 22.7 times greater risk for lower extremity amputation [5]. An analysis of Medicare data also found that diabetics with End Stage Renal Disease (ESRD) are ten times more likely to require amputations compared to diabetics without ESRD [6]. These findings raise the question of whether similar preexisting medical conditions increase the risk of traumatic lower extremity amputations as well. 

When analyzing traumatic amputations specifically, the Lower Extremity Assessment Project (LEAP) found that soft tissue condition was the best predictor of whether a limb was salvageable [7]. The LEAP Study also reported that a multitude of preexisting social, economic, and personality disadvantages contributed to poor outcomes. A recent study of patients with infection related to lower extremity fracture found that patients with CKD, intra-operative blood transfusions, surgical debridement, and gram-negative wound infection were significantly more likely to require amputation [8]. Additionally, patients with traumatic lower extremity fractures who contracted methicillin-resistant *Staphylococcus aureus *wound infections were more likely to require amputations than those with methicillin-sensitive *Staphylococcus aureus *[8]. Further research is required to determine whether these medical and social characteristics can be attributed to traumatic amputations in generalized populations as opposed to specific subgroups. 

Many patients who present with severe limb trauma may also have suffered a traumatic brain injury (TBI) as well. Current research is exploring links between head injury and bone repair. One case-control study determined that patients with a concurrent TBI and femoral fracture had significantly elevated serum levels of inflammatory cytokines, growth factors, and calcium in blood to accelerate the healing process [9]. Another study identified that the disruption of the blood-brain barrier from a TBI contributes to the release of thrombin, which stimulates osteoblast proliferation and inhibits apoptosis [10]. 

It is well known that alcohol and illicit drug use can lower inhibitions and impair motor functions. However, there has been little investigation into how substance abuse contributes to severe orthopedic trauma. A retrospective analysis of 12,857 patients enrolled in the Canadian Hospitals Injury Reporting and Prevention Program (CHIRPP) yielded interesting results regarding how drug and alcohol intoxication correlated with acute trauma causes. The study demonstrated that a greater proportion of the substance abuse group were treated for head/neck injuries, TBI, polytrauma, burns, and intentional injuries (violence or self-harm) [11]. Meanwhile, no association could be made linking acute substance abuse to motor vehicle trauma. A recent study was conducted on a subset of participants from the LEAP study to investigate the association between smoking and complications of the fracture healing process. While there are many confounding factors, they found that after adjusting for covariates, previous and current smokers have an increased time to union along with an increased likelihood of developing subsequent complications, including osteomyelitis [12]. Investigation of traumatic amputations may yield interesting results into the link between substance use, traumatic injuries, and patient outcomes.

The purpose of this study was to assess the medical characteristics associated with lower-extremity amputations resulting from trauma. While many factors, including the extent of soft tissue damage and vascular compromise, often necessitate acute traumatic amputations, this study seeks to determine whether previously described variables that contribute to non-traumatic amputations also apply to cases of limb trauma. Identifying risk factors for traumatic amputation can improve clinical decision-making and patient outcomes for both those undergoing amputations and those eligible for limb salvage. 

## Materials and methods

We created a retrospective lower extremity trauma database to perform our analysis, measuring several variables in traumatic lower extremity amputation patients at a level I trauma center in the United States to gain a comprehensive understanding of what other factors besides the initial injury may contribute to traumatic lower extremity amputation. Sixty-three patients with traumatic lower extremity amputations were identified via institutional billing records between 2017 and 2022 and placed into the study group. Only cases where amputation was directly attributable to lower extremity trauma were included. A chart review of the medical record was performed to ensure that billing records concurred with the care provided. Patients under the age of 18 were excluded. Patients who underwent amputation due to chronic illnesses unrelated to a prior history of trauma were excluded. Patients with missing documentation of important study variables were excluded. A control group of 129 lower extremity fracture patients who did not require amputation for similar injuries was isolated via billing records during that same period. Stratified sampling was used to ensure that demographics and trauma characteristics in the control group were similar to those in the study group. The control group was matched to the amputee group on average age (50.3 vs. 49.6 years), sex, race, mechanism of injury, and whether the fracture was open or closed. A chart review of the electronic health record of each patient was performed to record demographic data, medical history, trauma mechanism, injury severity, laboratory tests, and medical complications. After the necessary chart review, the data was stripped of all patient identifiers. The study was approved by an institutional review board at the authors’ university in compliance with 45 CFR 46, 45 CFR 164, 21 CFR 50, and 21 CFR 56.

Data was assessed for statistical significance using descriptives, Pearson chi-square tests, T tests, and ANOVA, as appropriate for the nominal or continuous level variables. Pearson chi-square tests were utilized to investigate the independence of two categorical variables, and ANOVA was selected to analyze three or more subgroups against a categorical variable. A 95% confidence level was used. Analyses were performed with SPSS 29.0 (IBM Corp., Armonk, USA). The level of statistical significance for all tests was set at p < 0.05.

## Results

Sixty-three traumatic lower extremity amputation patients were identified. Of these, 41 (65%) were male, and 46 (73%) of the amputations were below the knee (BKA); 56 (89%) of the amputation patients sustained complex injuries with multiple fractures, and 39 (62%) sustained polytraumas; and 39 patients (62%) also had injuries classified as high energy (gunshot wounds, motor vehicle accidents, and falls from a height greater than 10 feet). Motor vehicle accidents (MVA) and falls from a height of less than 10 feet were the most common injury mechanisms necessitating amputation. Thirty-six (57%) amputations were caused by MVA, and 19 (30%) were caused by falls from a height of less than 10 feet. Motorcycle accidents caused 16 (44%) of the traumatic amputations attributed to MVA.

**Figure 1:**
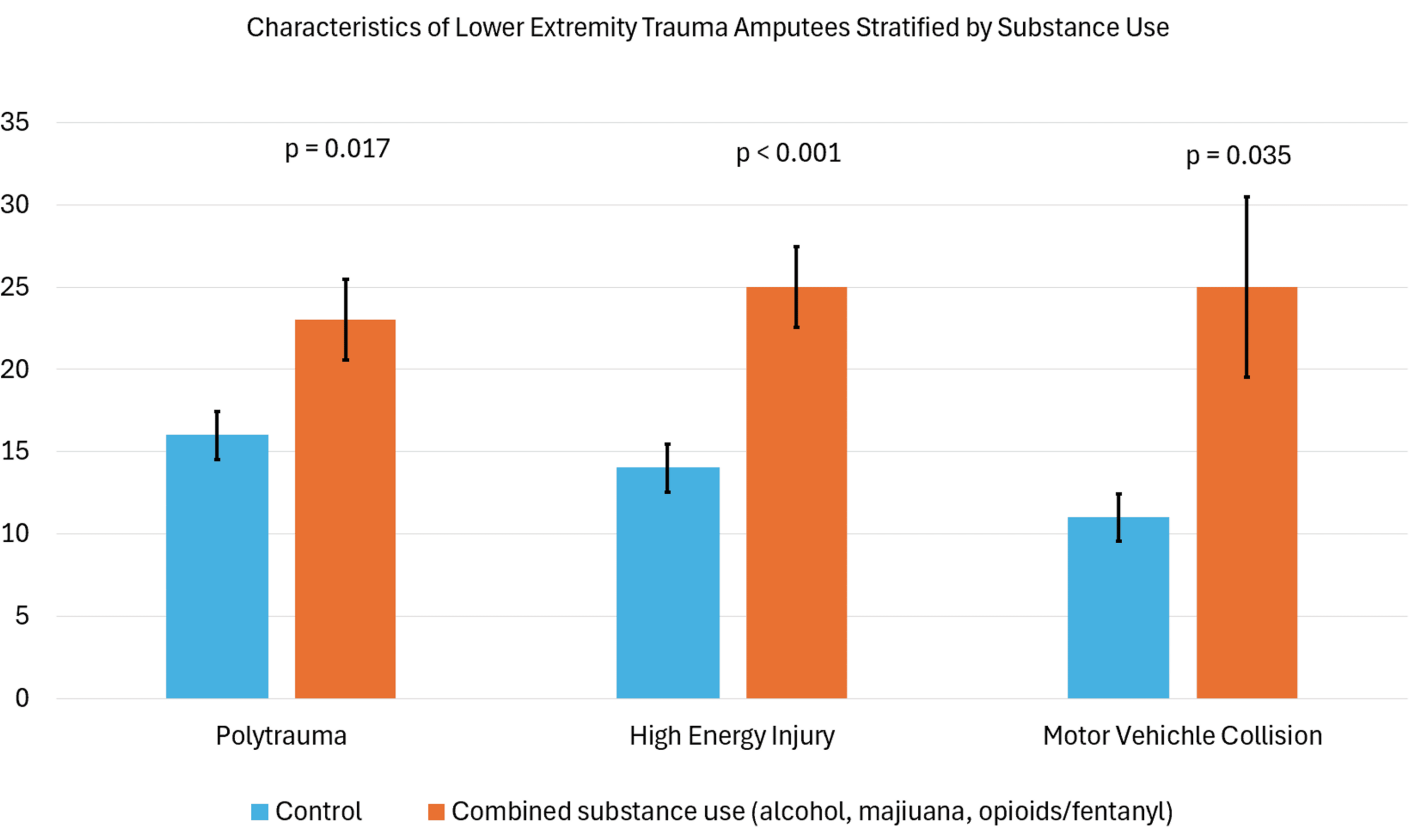
Clustered bar chart representing the rates of polytrauma, high-energy injury, and motor vehicle accidents for substance users and non-users who required lower extremity trauma amputation Error bars represent 95% confidence intervals. P-values from chi-square tests are provided. Significance was determined by p < 0.05.

Statistical analysis was first performed within the amputation group, revealing associations between substance use and higher injury severity. Among amputees, multi-system polytrauma, high-energy injuries, and MVA were associated with combined use of alcohol, marijuana, or opioids/fentanyl (Figure 1). Polytrauma remained associated with opioid/fentanyl use alone (p = 0.017). High energy injuries remained associated with opioid/fentanyl (p = 0.003), marijuana (p = 0.029), and methamphetamine (p = 0.011) use when analyzed individually. Statistical significance was not reached when analyzing alcohol or cocaine use alone. Surprisingly, combined use of alcohol, marijuana, or opioids/fentanyl was associated with significantly lower rates of fasciotomy (Table 1). This trend was preserved when analyzing alcohol users independently (p = 0.020). Furthermore, the data demonstrated interesting links between mechanisms of injury and substance use. Substance users were significantly more likely to receive injury due to motor vehicle trauma compared to non-users when all MVA subtypes were analyzed in aggregate (Table 2). 

**Table 1:** Characteristics of trauma lower extremity amputation patients stratified by combined substance use (alcohol, marijuana, or opioid/fentanyl) P-values for variables that surpassed the level of significance (p < 0.05) are bolded. Analysis was performed using chi-square tests.

	Combined Substance Use (n = 28)	Substance Not Used (n = 35)	Chi-square Value	P-value
Polytrauma	23	16	5.74	.017
High-Energy Injury	25	14	17.54	<0.001
Traumatic Brain Injury	8	6	1.73	0.630
Multiple Fractures	27	29	4.09	0.252
Fasciotomy	2	5	9.49	0.023

**Table 2:** Classification of motor vehicle accident of trauma lower extremity amputation patients stratified by combined substance use (alcohol, marijuana, or opioid/fentanyl) P-values for variables that surpassed the level of significance (p < 0.05) are bolded. Analysis was performed using chi-square tests.

	Combined Substance Use (n = 28)	Substance Not Used (n = 35)	Chi-square Value	P-value
Car Accident	6	2		
Motorcycle	10	7		
Pedestrian	5	0		
Train	1	1		
Agricultural/Industrial	1	1		
Ejection from vehicle	2	0		
Total	25	11	26.26	0.035

When all identified amputees were compared to the control group, many statistically significant associations were discovered (Table 3). Traumatic lower extremity amputations were more likely to occur in trauma patients with diabetes, hypertension, osteomyelitis, fasciotomy, CKD, acute alcohol intoxication, and opiates/fentanyl use. Patients who sustained TBI with their injury were less likely to require amputation, while patients with multiple fractures were more likely to undergo amputation. No significant differences were found in the rates of tobacco use and polytrauma between the two groups. To prevent tissue quality and acute vascular compromise from confounding these correlations, the amputation group was subdivided based on the time from injury to amputation. Patients who underwent amputation within 48 hours after trauma were classified as emergent amputations. Patients who underwent amputation on day three or later were classified as non-emergent. Sixteen of the amputations were identified as emergent, and statistical tests were performed again without these patients included. Despite this intervention, statistical significance was still preserved when comparing rates of diabetes, hypertension, osteomyelitis, fasciotomy, alcohol intoxication, and CKD between both groups (Table 4). Injuries with multiple fractures were more likely to result in amputation. TBI remained a protective factor against amputation in this analysis. Rates of opioid/fentanyl use, tobacco use, osteoporosis, and polytrauma between the two groups were not significantly different.

**Table 3:** Characteristics of patients presenting with lower extremity trauma stratified by those requiring lower extremity amputation P-values for variables that surpassed the level of significance (p < 0.05) are bolded. Analysis was performed using chi-square tests. TBI: traumatic brain injury; CKD: chronic kidney disease

	Control (n = 129)	Amputation (n = 63)	Chi-square Value	P-value
Diabetes	24	24	8.58	0.003
Hypertension	61	49	16.08	<0.001
Alcohol Intoxication	46	37	9.18	0.002
Tobacco Use	81	42	0.276	0.599
Opioid/Fentanyl	19	19	6.35	0.012
Osteomyelitis	5	21	31.37	<0.001
Osteoporosis	25	11	0.102	0.749
Fasciotomy	4	11	12.12	<0.001
Polytrauma	62	39	3.25	0.071
TBI	55	15	6.48	0.011
CKD	9	13	7.78	0.005
Multiple Fractures	87	56	10.24	0.001

**Table 4:** Characteristics of patients presenting with lower extremity trauma stratified by those requiring lower extremity amputation more than 48 hours after initial injury P-values for variables that surpassed the level of significance (p < 0.05) are bolded. Analysis was performed using chi-square tests. TBI: traumatic brain injury; CKD: chronic kidney disease

	Control (n = 129)	Non-emergent Amputation (n = 47)	Chi-square Value	P-value
Diabetes	24	22	14.19	<0.001
Hypertension	61	40	20.15	<0.001
Alcohol Intoxication	46	26	5.51	0.019
Tobacco Use	81	32	0.420	0.517
Opioid/Fentanyl	19	9	0.503	0.478
Osteomyelitis	5	20	42.92	<0.001
Osteoporosis	25	11	0.343	0.558
Fasciotomy	4	10	15.54	<0.001
Polytrauma	62	26	0.726	0.394
TBI	55	10	6.75	0.009
CKD	9	11	9.23	0.002
Multiple Fractures	87	40	5.35	0.021

Further attention was then directed towards the use of lower extremity CT angiography (CTA) and documented dorsalis pedis pulses within the amputation group (Table 5). Nine patients with a palpable dorsalis pedis underwent CTA (21%), compared to 10 patients without a palpable dorsalis pedis on the affected limb who underwent CTA (48%). This demonstrated that pulseless patients were more likely to undergo CTA before surgery. When viewing the emergent amputations in isolation, only three patients had palpable dorsalis pedis pulses on presentation. While roughly half of patients without a dorsalis pedis pulse in this group received a CTA, statistical significance was not reached due to low sample size. An analysis of non-emergent amputations did not reveal an increased use of CTA compared to the emergent group.

**Table 5:** Comparing rates of lower extremity CT angiography in patients requiring delayed lower extremity amputation stratified by the absence of a dorsalis pedis pulse on the affected leg P-values for variables that surpassed the level of significance (p < 0.05) are bolded. Analysis was performed using chi-square tests. CTA: lower extremity computed tomography angiography

		Dorsalis Pedis Present	No Pulse Present	Chi-square Value	P-value
All Amputations (n = 63)	CTA performed	9	10		
	No CTA	33	11		
	Total	42	21	4.56	0.033
Emergent Amputations (n = 16)	CTA performed	2	6		
	No CTA	1	7		
	Total	3	13	0.410	0.552
Non-emergent Amputations (n = 47)	CTA performed	7	4		
	No CTA	32	4		
	Total	39	8	3.80	0.051

We then compared amputations that occurred within 48 hours of injury to amputations that were performed beyond that initial period (Table 6). While patients with multiple fractures and high-energy injuries were not significantly associated with emergent amputation, osteoporosis was significantly associated with non-emergent amputations. All 11 of the patients with osteoporosis underwent amputation more than 48 hours after injury. While osteoporosis was not associated with more frequent amputations compared to the control group, it appears that bone quality (osteoporosis) may indicate a worse prognosis for limb salvage over time among the amputee group. However, this relationship may be confounded by the age, frailty, and general health of soft tissue in the injured limb, in addition to bone quality.

**Table 6:** Characteristics of patients presenting with trauma resulting in lower extremity amputation stratified by whether amputation occurred within the first 48 hours after injury P-values for variables that surpassed the level of significance (p < 0.05) are bolded. Analysis was performed using chi-square tests. TBI: traumatic brain injury

	Emergent Amputation (n = 16)	Non-emergent Amputation (n = 47)	Chi-square Value	P-value
TBI	5	10	0.655	0.419
Multiple Fractures	16	40	2.68	0.102
Osteoporosis	0	11	4.54	0.033

## Discussion

This study provides valuable insights into the factors associated with traumatic lower extremity amputations, emphasizing the role of pre-existing medical conditions. Our initial analysis of amputee data highlights the negative impact of drug and alcohol use on traumatic injuries. This study confirms a relationship between substance use and higher energy injuries and polytraumas, resulting in traumatic lower extremity amputation. MVA was the most common trauma mechanism resulting in lower extremity amputation, and substance users were more likely to experience unsalvageable limb trauma from MVA compared to non-users. 

While previous studies have not found an association between substance use and MVA trauma, the ubiquitous nature of automobile travel in developed nations may account for the fact that overall trauma from an MVA is not associated with substance use. However, our research demonstrates that among the most severe trauma cases - those requiring amputation - substance users were more likely to sustain high-energy polytraumas from motor vehicle-related injuries. While it is possible that substance use contributed to lower rates of fasciotomy and compartment syndrome, it is more likely that these severe polytrauma cases underwent emergent amputation, rather than attempting a limb salvage requiring a fasciotomy.

**Figure 2:**
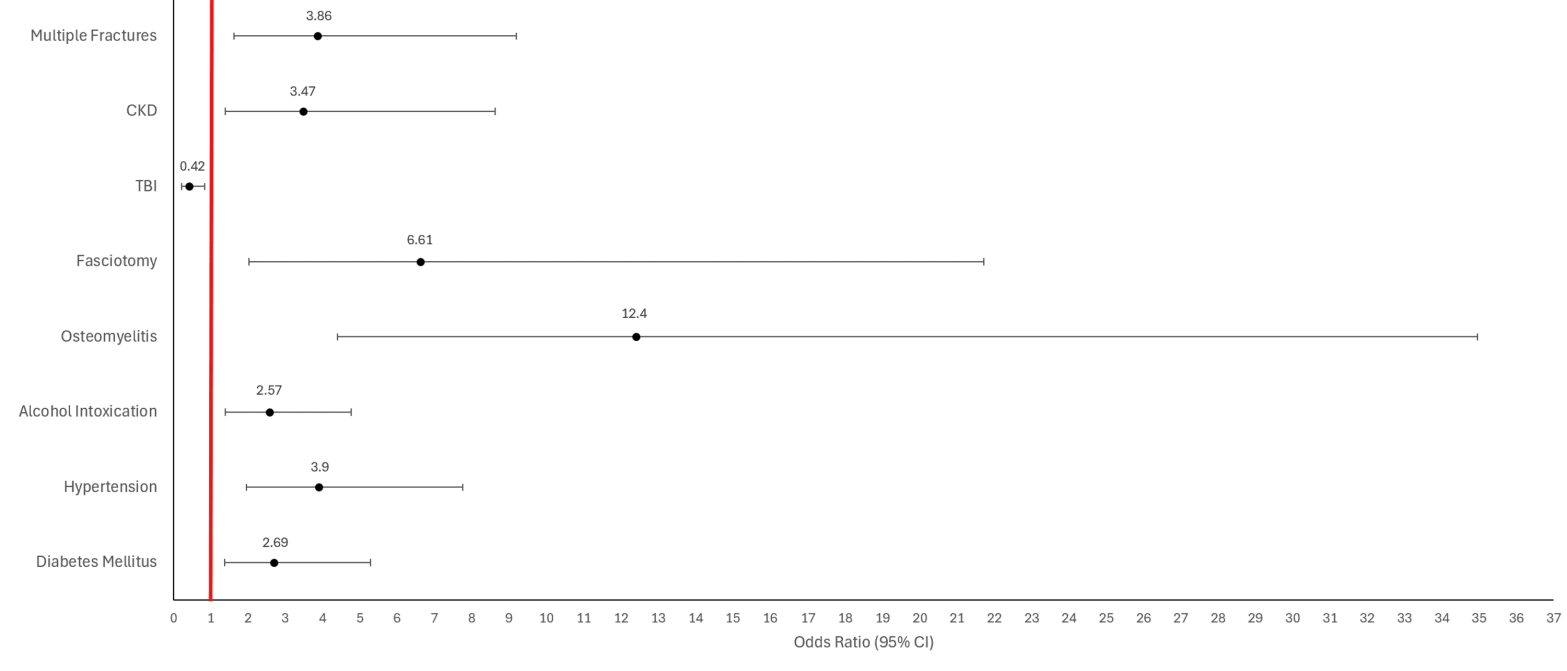
Forest plot providing odds ratios for exposure to various comorbidities and an outcome of traumatic lower extremity amputation Error bars represent 95% confidence intervals. The line of no effect is set at a value of one. Odds ratios greater than 1 suggest that a comorbidity is associated with an increased risk of amputation. TBI has an odds ratio less than 1, demonstrating an inverse relationship between exposure to TBI and the outcome of amputation. TBI was associated with a 58% reduction in risk of amputation. Analysis was conducted with a sample size of all 63 amputations studied. CI: confidence interval; CKD: chronic kidney disease; TBI: traumatic brain injury

**Figure 3:**
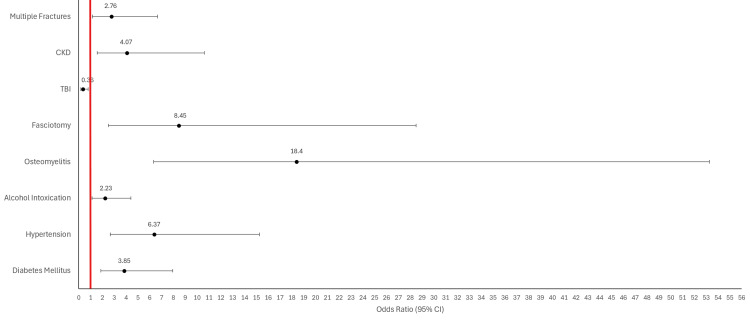
Forest plot providing odds ratios for exposure to various comorbidities and an outcome of traumatic lower extremity amputation occurring greater than 48 hours after injury Error bars represent 95% confidence intervals. The line of no effect is set at a value of one. Odds ratios greater than 1 suggest that a comorbidity is associated with an increased risk of amputation. TBI has an odds ratio less than 1, demonstrating an inverse relationship between exposure to TBI and the outcome of amputation. TBI was associated with a 64% reduction in risk of amputation. Analysis was conducted with a sample size of 47 amputations that were performed more than 48 hours after presentation to the emergency department. CI: confidence interval; CKD: chronic kidney disease; TBI: traumatic brain injury

A plethora of previous research has been done to document the connection between underlying medical conditions and chronic amputations [13]. However, our research reveals that these associations persist with traumatic amputations. Specifically, we found that lower extremity trauma patients with comorbidities such as diabetes, hypertension, osteomyelitis, fasciotomy, acute alcohol intoxication, and CKD were significantly more likely to require amputation after suffering a lower extremity trauma (Figure 2). Each of these conditions is known to contribute to decreased perfusion or slowed wound healing, and the presence of ethanol in blood can also impair platelet activity, accelerating blood loss [14]. Significance was preserved even after excluding emergent amputations, which provided for improved matching of variables with the control group and excluded instances where extensive trauma or vascular compromise played an overriding role in thwarting limb salvage efforts (Figure 3). Patients suffering multiple lower extremity fractures were still associated with amputation after excluding emergent amputations, but this relationship was less strong after excluding emergent amputations. A lack of a significant correlation between polytrauma and amputation provides additional evidence that our control group did not skew towards less severe injuries. The odds ratios of these variables quantify the increased risk of amputation due to exposure to preexisting comorbidities. Studies with larger sample sizes will yield tighter confidence intervals, providing greater certainty of these risks. The persistence of significant associations between these medical comorbidities and amputation - regardless of the urgency of the procedure - suggests that medical decision-making in limb salvage should account for a patient’s medical history across multiple organ systems. These associations could also impact the management of comorbid conditions in cases of limb trauma. Future research could lead to more concrete guidelines for glycemic control, blood pressure management, or antibiotic prophylaxis in cases where trauma threatens limb loss. Complex algorithms and programs can also be theorized that account for the medical and social comorbidities of limb trauma patients to calculate amputation risk preoperatively. 

Our research found that patients with traumatic brain injuries were significantly less likely to undergo amputation. TBI was associated with a 58% reduction in risk of amputation when all amputations were studied, and a 64% reduction in risk of amputation when emergent cases were excluded. The concept of TBI as a protective factor may seem counterintuitive, but this finding concurs with previous research that links TBI to improved fracture healing time [9,10]. With additional corroborating evidence, physicians may view TBI as an important prognostic factor for limb salvage candidates in the future, especially in cases of delayed amputation where fracture healing/non-union may play a greater role. 

When analyzing the usage of CT angiography as a diagnostic tool in patients with lower extremity trauma requiring amputation, we found that patients without a dorsalis pedis pulse were significantly more likely to undergo CTA. However, these associations were not maintained in our subgroup analysis. This finding raises interesting questions about the utility of CTA when limb salvage is in question. Our results point to the need for future research, which can provide more concrete guidelines for physicians on the proper usage of diagnostic imaging in these scenarios to provide high-value care. 

Interestingly, our entire cohort of amputees with osteoporosis underwent delayed amputation following a traumatic event. Osteoporosis was not linked to amputation outright, but it is plausible that decreased bone density contributes to prolonged healing times and complicates surgical repair. Osteoporosis may be a general indicator for non-union and delayed callous formation [15]. This observation aligns with our hypothesis that underlying disease states increase the likelihood of amputation in traumatic scenarios. Additional analysis with larger samples could provide better clarity on this question with tight control for confounding variables.

The results from this study reinforce the importance for medical providers to obtain a robust history and physical exam of trauma patients and highlight the necessity for providers to take a complete social history to counsel patients about substance use. Our research provides an initial investigation into how medical comorbidities contribute to the likelihood of lower extremity amputation after traumatic injury. With future validation, screening for specific comorbidities in trauma settings may be recommended to predict amputation risk. Public health officials may also find this data useful as it pertains to motor vehicle injury, especially as more US cities and states consider drug decriminalization and legalization policies. MVA was the most common injury mechanism necessitating amputation, and alcohol intoxication was associated with increased rates of amputation, further demonstrating the risk of operating machinery under the influence. 

Our research is limited by a relatively small sample size, and further research should be conducted with larger trauma databases to confirm the conclusions drawn from this dataset. While the patients included in this case-control study received care at a single level I trauma center, demographics and regional variations may confound our findings, and it is possible that our data does not reflect trends found within the general population. 

Larger sample sizes will provide more opportunities for subgroup analyses, particularly for stratifying injury severity scores. We recognize that soft tissue injury greatly influences whether limb salvage is possible and attempted to control for this possible confounding variable. In this study, amputees were matched to fracture controls based on whether fractures were classified as open or closed. This was done in order to prevent severe soft tissue trauma from confounding the dataset. While the severity of open fracture wounds can be more precisely subdivided using the Gustilo-Anderson classification system, we felt that using this method to match patients would decrease the efficacy of this study, given a relatively small sample size. Splitting the amputation group into Gustilo-Anderson subgroups would create smaller samples to perform statistical analysis on. Additionally, low rates of inter-observer agreement in Gustilo-Anderson scoring created doubt that any associations linked to these classifications would be reliable [16]. Matching patients based on whether the fracture created an open wound preserved the viability of the sample size and provided a simple and objective metric for ensuring that injury severity was grossly similar between the amputation group and the controls. Future studies with large databases can be directed toward matching patients on open fracture severity scores specifically.

While our data covered a variety of injury types, this sample could not account for all conceivable trauma mechanisms in adequate numbers. Toxicology screening and verbalized histories obtained at the time of injury were used to determine patients who were substance users, but misidentification is theoretically possible. Our research was also limited to lower extremity trauma. It is possible that various medical comorbidities interact differently in upper extremity trauma, as the upper extremities typically have a more robust vascular supply. Furthermore, given the crucial role of infection in limb salvage decisions, further studies centered on specific fracture-related infections may correlate more strongly with amputation risk and further contextualize our findings. Future research can be focused on expanding the size of our current dataset, directly assessing tissue quality in open fractures, and ensuring that patient variation is reflective of larger populations. 

## Conclusions

Causal relationships have been previously established between many medical comorbidities and chronic amputation. This case-control study performed at a single-level I trauma center demonstrated that pre-existing comorbidities - including diabetes, hypertension, CKD, osteomyelitis, fasciotomy, and acute alcohol intoxication - are significantly associated with an increased risk of traumatic lower extremity amputation. These associations persisted even when emergent amputations performed within 48 hours of injury were excluded from the analysis. Conversely, exposure to TBI was associated with limb salvage, supporting prior evidence of its role in promoting fracture healing. Among lower extremity traumas requiring amputation, active substance users were more likely to suffer high-energy polytraumas and present after a MVA compared to patients who were not under the influence of drugs or alcohol. These findings underscore the importance of integrating a patient’s medical and social history into clinical decision-making for limb salvage. Larger, multi-center studies are warranted to validate these associations and inform evidence-based guidelines for optimizing outcomes in traumatic lower extremity injuries.
